# Combined exposure of diesel exhaust particles and respirable Soufrière Hills volcanic ash causes a (pro-)inflammatory response in an in vitro multicellular epithelial tissue barrier model

**DOI:** 10.1186/s12989-016-0178-9

**Published:** 2016-12-12

**Authors:** Ines Tomašek, Claire J. Horwell, David E. Damby, Hana Barošová, Christoph Geers, Alke Petri-Fink, Barbara Rothen-Rutishauser, Martin J. D. Clift

**Affiliations:** 1 Institute of Hazard, Risk and Resilience, Department of Earth Sciences, Durham University, Science Labs, Durham, DH1 3LE UK; 2 BioNanomaterials, Adolphe Merkle Institute, University of Fribourg, Chemin des Verdiers 4, CH-1700 Fribourg, Switzerland; 3 Department of Earth and Environmental Sciences, Section for Mineralogy, Petrology and Geochemistry, Ludwig-Maximilians-Universität München, Theresienstrasse 41, 80333 Munich, Germany; 4 In Vitro Toxicology Group, Institute of Life Sciences, Swansea University Medical School, Singleton Park Campus, Swansea, SA2 8PP Wales UK; 5 Chemistry Department, University of Fribourg, Chemin des Musee, CH-1700 Fribourg, Switzerland; 6 United States Geological Survey, 345 Middlefield Road, Menlo Park, CA 94025 USA

**Keywords:** Volcanic ash, Diesel exhaust particles, In vitro, Particle co-exposures, Multicellular Human Epithelial Tissue Barrier System, Air-liquid Interface Exposures, (pro-)inflammatory cytokines/chemokines

## Abstract

**Background:**

There are justifiable health concerns regarding the potential adverse effects associated with human exposure to volcanic ash (VA) particles, especially when considering communities living in urban areas already exposed to heightened air pollution. The aim of this study was, therefore, to gain an imperative, first understanding of the biological impacts of respirable VA when exposed concomitantly with diesel particles.

**Methods:**

A sophisticated in vitro 3D triple cell co-culture model of the human alveolar epithelial tissue barrier was exposed to either a single or repeated dose of dry respirable VA (deposited dose of 0.26 ± 0.09 or 0.89 ± 0.29 μg/cm^2^, respectively) from Soufrière Hills volcano, Montserrat for a period of 24 h at the air-liquid interface (ALI). Subsequently, co-cultures were exposed to co-exposures of single or repeated VA and diesel exhaust particles (DEP; NIST SRM 2975; 0.02 mg/mL), a model urban pollutant, at the pseudo-ALI. The biological impact of each individual particle type was also analysed under these precise scenarios. The cytotoxic (LDH release), oxidative stress (depletion of intracellular GSH) and (pro-)inflammatory (TNF-α, IL-8 and IL-1β) responses were assessed after the particulate exposures. The impact of VA exposure upon cell morphology, as well as its interaction with the multicellular model, was visualised *via* confocal laser scanning microscopy (LSM) and scanning electron microscopy (SEM), respectively.

**Results:**

The combination of respirable VA and DEP, in all scenarios, incited an heightened release of TNF-α and IL-8 as well as significant increases in IL-1β, when applied at sub-lethal doses to the co-culture compared to VA exposure alone. Notably, the augmented (pro-)inflammatory responses observed were not mediated by oxidative stress. LSM supported the quantitative assessment of cytotoxicity, with no changes in cell morphology within the barrier model evident. A direct interaction of the VA with all three cell types of the multicellular system was observed by SEM.

**Conclusions:**

Combined exposure of respirable Soufrière Hills VA with DEP causes a (pro-)inflammatory effect in an advanced in vitro multicellular model of the epithelial airway barrier. This finding suggests that the combined exposure to volcanic and urban particulate matter should be further investigated in order to deduce the potential human health hazard, especially how it may influence the respiratory function of susceptible individuals (i.e. with pre-existing lung diseases) in the population.

**Electronic supplementary material:**

The online version of this article (doi:10.1186/s12989-016-0178-9) contains supplementary material, which is available to authorized users.

## Background

With nearly 10% of the world’s population living near a historically active volcano [1], there is long-standing concern over the capacity of respirable-sized volcanic ash (VA) to cause acute and chronic respiratory health effects [2]. Substantial knowledge of the posed respiratory hazard, alongside extensive characterisation of the physicochemical properties of respirable VA, has been obtained in recent years [3, 4], leading to a better understanding of the structure-toxicity relationship [5]. However, with many volcanoes situated near large cities, VA is rarely inhaled in isolation; instead, VA is commonly exposed to the human population concomitantly with additional substances, notably anthropogenic pollution. A prime example of this is Mexico City, which was named by the United Nations as the world’s most polluted city in 1992 [6] and sits just 70 km from the frequently-erupting Popocatepetl volcano.

Exposure to anthropogenic pollution is strongly associated with adverse health effects, predominantly pulmonary and cardiovascular diseases, as well as reduced respiratory health [7–12]. The human population resident in urban areas is particularly affected by high levels of anthropogenic particulate matter (PM) since vehicles are primary emitters of PM, with diesel exhaust particles (DEP) being the main constituent [13]. Yet, currently, limited understanding surrounds the human health hazard associated with the combined exposures (i.e. inhalation) that results from the addition of volcanic PM to the urban environment. Of particular importance is the consideration of how respirable VA may interact with DEP and how this may contribute, or not, to a heightened, potential respiratory hazard.

The aim of the present study, therefore, was to investigate the biological impact of a concomitant exposure to VA (Soufrière Hills volcano, Montserrat) and a standardised DEP sample (*National Institute of Standards and Technology’s Standard Reference Material* (NIST SRM) 2975)) [14] for the first time, using an established, advanced multicellular in vitro model mimicking the human epithelial tissue barrier [15]. The basis for this project stemmed from the British Geological Survey’s report to the UK Government on characteristics of a future large, effusive Icelandic eruption, which highlighted the urgent need to evaluate the role of mixing volcanic emissions with anthropogenic pollutants and whether this would affect the individual respiratory hazard of either particle independently [16]. Thus, the current study provides a landmark first assessment of these issues, the findings of which are highly relevant for volcanic health hazard management on a global scale.

## Results

### Particle characterisation

Particle size analysis of an isolated respirable fraction from the Soufrière Hills ash sample MVO12/7/03 showed that all particles were <10 μm. The sample consisted of 12.2, 41.5 and 72.5% volume of particles with sizes of <1, <2.5 and <4 μm, respectively (Additional file 1). Specific surface area, as determined by the Brunauer-Emmett-Teller (BET) [17] analysis with nitrogen adsorption, was 3.2 m^2^/g.

Characteristics for the NIST SRM 2975 used were previously reported in [18]. Briefly, DEP exhibited a mean diameter of 1.62 μm (denoted by number distribution). DEP specific surface area was 91 m^2^/g, as determined by BET with nitrogen adsorption.

### Nebulisation of VA

**Fig. 1:**
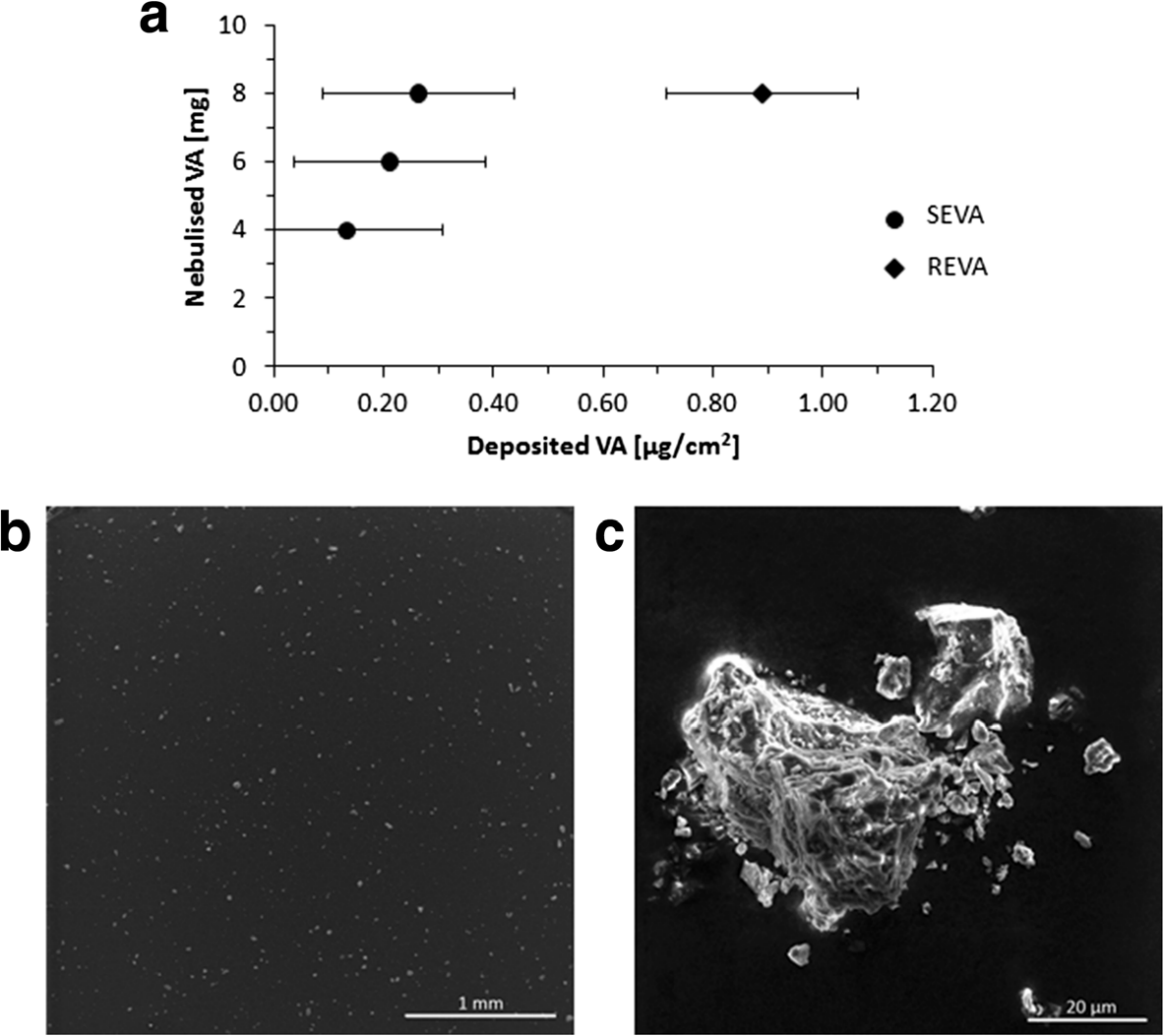
Deposition of nebulized respirable fraction of volcanic ash. **a** Average mass deposition (μg/cm^2^) of volcanic ash (VA) quantified using a quartz crystal microbalance (QCM), following nebulisation of dry respirable ash (*MVO12/7/03*) using a dry powder insufflator (DP-4*, Penn Century, USA*) under the following conditions: single exposure (SEVA) with 4 mg (*n* = 14), 6 mg (*n* = 14) or 8 mg (*n* = 17), as well as repeated exposure (REVA) to 8 mg (nebulised 3× within 15 min; *n* = 9). Data are presented as the mean ± standard error of the mean. Scanning electron micrographs of nebulized, uncoated ash sample (REVA), show **b** heterogeneous particle dispersion (WD: 5.53 mm, MAG: 97×) and **c** an inset of image (**b**) (WD: 7 mm, MAG: 3.80 k ×). Images were collected at 10 kV. Scale bars are 1 mm (**b**) and 20 μm (**c**)

A dry powder insufflator (DP-4, *Penn Century, USA*) was used to nebulise the respirable fraction of the VA for direct deposition onto the in vitro lung cell culture at the air-liquid interface (ALI). As this was the first study to administer VA at the ALI, an initial dose-dependent analysis of the VA deposition was conducted to determine cell-delivered dose, as well as its biological impact at these different doses. The cell-delivered dose was monitored using an integrated quartz crystal microbalance (QCM) and showed a concentration-dependent deposition of the VA sample (Fig. 1a). The average deposited doses were 0.13 ± 0.03, 0.21 ± 0.06, 0.26 ± 0.09 and 0.89 ± 0.29 μg/cm^2^ (relative to an administered mass of 4, 6, 8 mg and a repeated administered mass of 8 mg, respectfully). The threshold limit for the QCM was 0.09 μg/cm^2^. Scanning electron microscopy (SEM) imaging of the nebulised respirable ash sample (0.89 ± 0.29 μg/cm^2^) revealed a heterogeneously dispersed deposition of ash particles (Fig. 1b).

### Particle-cell exposures

#### Volcanic ash

An initial dose-dependent analysis of cytotoxicity, oxidative stress potential and (pro-)inflammatory response using administered VA masses of 4, 6 and 8 mg (Additional files 2 and 3) indicated all doses to be sub-lethal to the co-culture system. Due to the reliability, as well as a greater and effective deposition of the highest administered mass, it was subsequently used to assess the biological impact of VA as either a single exposure (SEVA; 0.26 ± 0.09 μg/cm^2^) or repeated exposure (REVA; 0.89 ± 0.29 μg/cm^2^) towards the in vitro triple cell co-culture model of the epithelial tissue barrier.

**Fig. 2:**
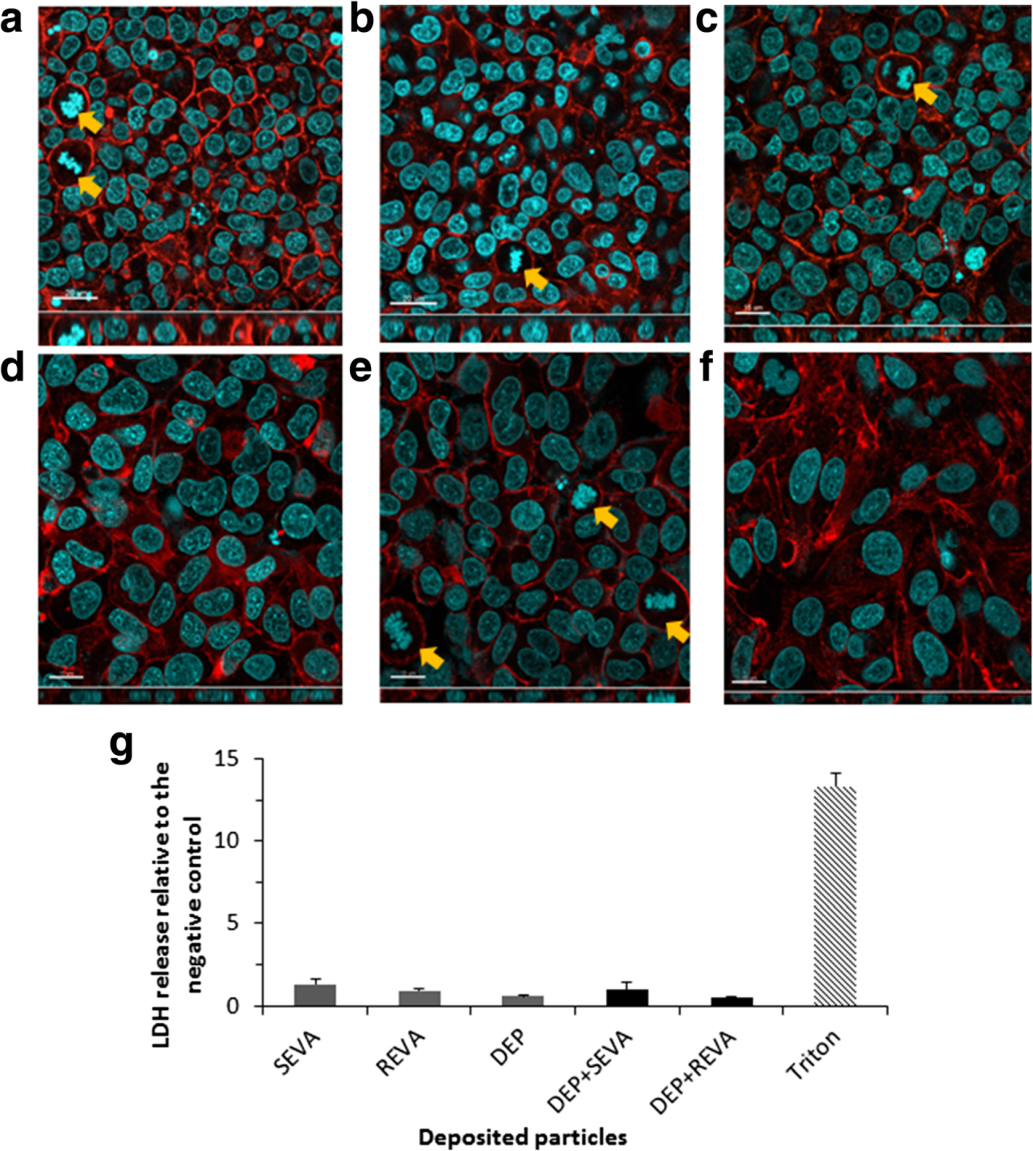
Cell morphology and cytotoxicity of triple cell co-cultures exposed to volcanic ash and diesel exhaust particles. Confocal laser scanning microscopy (LSM) images show the complete triple cell co-culture (i.e. A549 type-II ‘like’ epithelial cell monolayer with human blood monocyte macrophages (MDM) and dendritic cells (MDDC) on the apical and basal sides, respectively) stained for F-actin cytoskeleton (*red*) and the nuclei (*blue*). **a** Control and cultures exposed to **b** 0.26 ± 0.09 μg/cm^2^ of single exposure to volcanic ash (SEVA), **c** 0.89 ± 0.29 μg/cm^2^, repeated exposure to volcanic ash (REVA), **d** diesel exhaust particles (DEP; 0.02 mg/mL), **e** diesel exhaust particles and 0.26 ± 0.09 μg/cm^2^ of single exposure to volcanic ash (DEP + SEVA), and **f** diesel exhaust particles and 0.89 ± 0.29 μg/cm^2^ of repeated exposure to volcanic ash (DEP + REVA). Yellow arrows indicate cells undergoing cell division. Scale bars are 20 μm (**a**-**b**) and 15 μm (**c**-**f**). Images were collected at magnification 63×. **g** Cytotoxicity determined by the release of lactate dehydrogenase (LDH) from the triple cell co-culture following exposure to SEVA, REVA, DEP, DEP + SEVA and DEP + REVA. Data are presented as fold increase relative to the negative control (supplemented cell culture medium only) ± standard error of the mean. Triton X-100 at 0.2% in phosphate buffered saline (PBS) acted as the positive assay control. LDH data shown are related to the following repetitions for each exposure: SEVA *n* = 4; REVA, DEP, DEP + SEVA and DEP + REVA *n* = 3; negative and positive controls *n* = 8

**Fig. 3:**
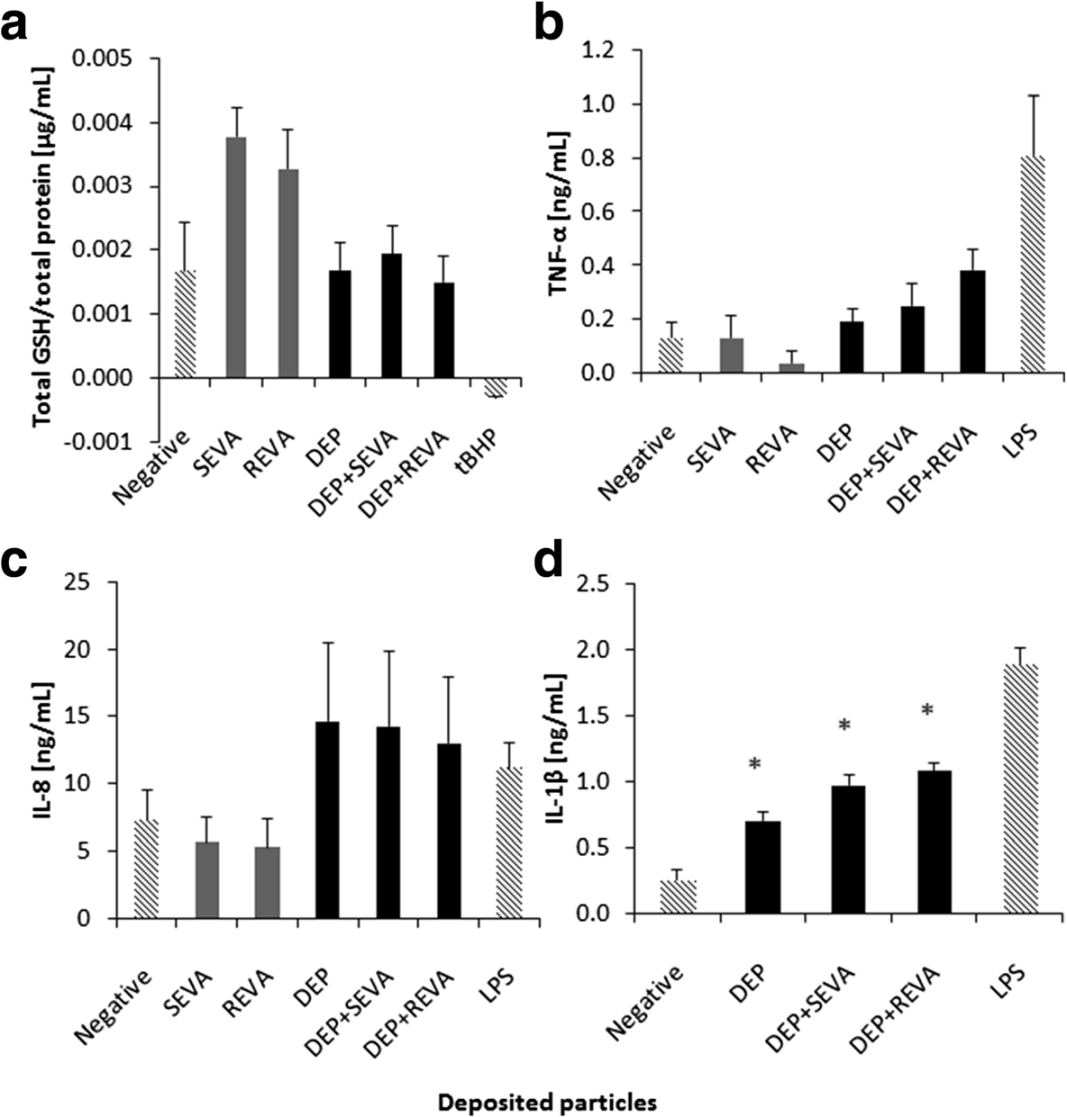
Biochemical response of triple cell co-culture system following exposures to volcanic ash and diesel exhaust particles. **a** Total reduced glutathione (GSH), **b** tumour necrosis factor-α (TNF-α) release, **c** interleukin-8 (IL-8) release and **d** interleukin-1β (IL-1β) release of the triple cell co-culture model after exposure to 0.26 ± 0.09 μg/cm^2^ of single exposure to volcanic ash (SEVA), 0.89 ± 0.29 μg/cm^2^ of repeated exposure to volcanic ash (REVA), diesel exhaust particles (DEP; 0.02 mg/mL), co-exposure to diesel exhaust particles and 0.26 ± 0.09 μg/cm^2^ of single exposure to volcanic ash (DEP + SEVA), and co-exposure to diesel exhaust particles and 0.89 ± 0.29 μg/cm^2^ of ﻿repeated exposure﻿ to﻿ volcanic ash (DEP + REVA). The respective positive assay controls are *tert*-Butyl Hydrogen Peroxide (*t*BHP; 250 μL of 100 mM) and lipopolysaccharide (LPS; 100 μL of 1 μg/mL), added to the apical and basal compartment of the triple cell co-culture, respectively. The negative control was cell culture medium only. Data are presented as the mean ± standard error of the mean. Data shown are related to the following repetitions for each exposure: SEVA *n* = 4; REVA, DEP, DEP + SEVA and DEP + REVA *n* = 3; negative and positive controls *n* = 8. * indicates *p* < 0.05

As determined *via* release of the cytosolic enzyme lactate dehydrogenase (LDH), no significant cytotoxicity (*p* > 0.05) was observed after 24 h following exposure to either SEVA or REVA compared to the negative control (defined as supplemented cell culture medium with no particle exposure) (Fig. 2g). The lack of any cytotoxicity associated with the SEVA and REVA exposures upon the cell cultures was qualitatively supported by the observation that no alteration to cellular morphology occurred, as visualised by laser scanning microscopy (LSM) (Fig. 2b and c). It was further observed, by LSM, that the epithelial layer was tightly bound together, forming a monolayer, with cells undergoing mitosis, suggestive of normal homeostasis (Fig. 2b and c). Further assessment of the biochemical impact of SEVA and REVA upon the triple cell co-culture showed no significant (*p* > 0.05) loss in total reduced glutathione (GSH), a key indicator of oxidative stress in vitro [19] (Fig. 3a). Similar, negative effects were also observed for the ability of either SEVA or REVA to elucidate a (pro-)inflammatory response, with no significant (*p* > 0.05) production of tumour necrosis factor-α (TNF-α) or interleukin-8 (IL-8) after 24 h exposure (Fig. 3b and c). It is important to note that, alongside these negative datasets, all positive controls used (i.e. *tert*-Butyl Hydrogen Peroxide (*t*BHP; GSH assay) and lipopolysaccharide (LPS; TNF-α and IL-8)) caused significant increases within the respective biological marker, indicating that the biological model used was responsive for all assay endpoints measured.

#### Diesel exhaust particles

Similar findings were observed following exposure of the co-culture to DEP alone, with no significant cytotoxicity (Fig. 2d and g) or changes (*p* > 0.05) to the oxidative stress status of cells observed (Fig. 3a). Importantly, the dose used of DEP was based upon the findings previously shown by Clift et al. [20], who undertook a dose-dependent analysis of the same DEP sample upon the same co-culture system. Again, the positive assay control, *t*BHP, showed a significant depletion of GSH in the co-culture system, confirming the observation that the array of sample exposures incited no oxidative stress. Despite these findings, the DEP only exposures to the in vitro multicellular system elicited a significant (*p* < 0.05) interleukin-1β (IL-1β) and non-significant (*p* > 0.05) TNF-α and IL-8 responses compared to the negative control (Fig. 3b and c).

#### Combined exposure of SEVA and REVA with DEP

Combined exposures to DEP and VA (DEP + SEVA, DEP + REVA) also resulted in no significant cytotoxicity (Fig. 2e, f and g) or changes (*p* > 0.05) to the oxidative stress status of cells (Fig. 3a). It was observed, however, that although the combined exposures did induce an heightened (pro-)inflammatory response in the co-cultures for TNF-α and IL-8 (*p* > 0.05), only a significant (*p* < 0.05) release of IL-1β, compared to the negative control, was noted (Fig. 3b–d).

### Interaction of VA with triple cell co-cultures

**Fig. 4:**
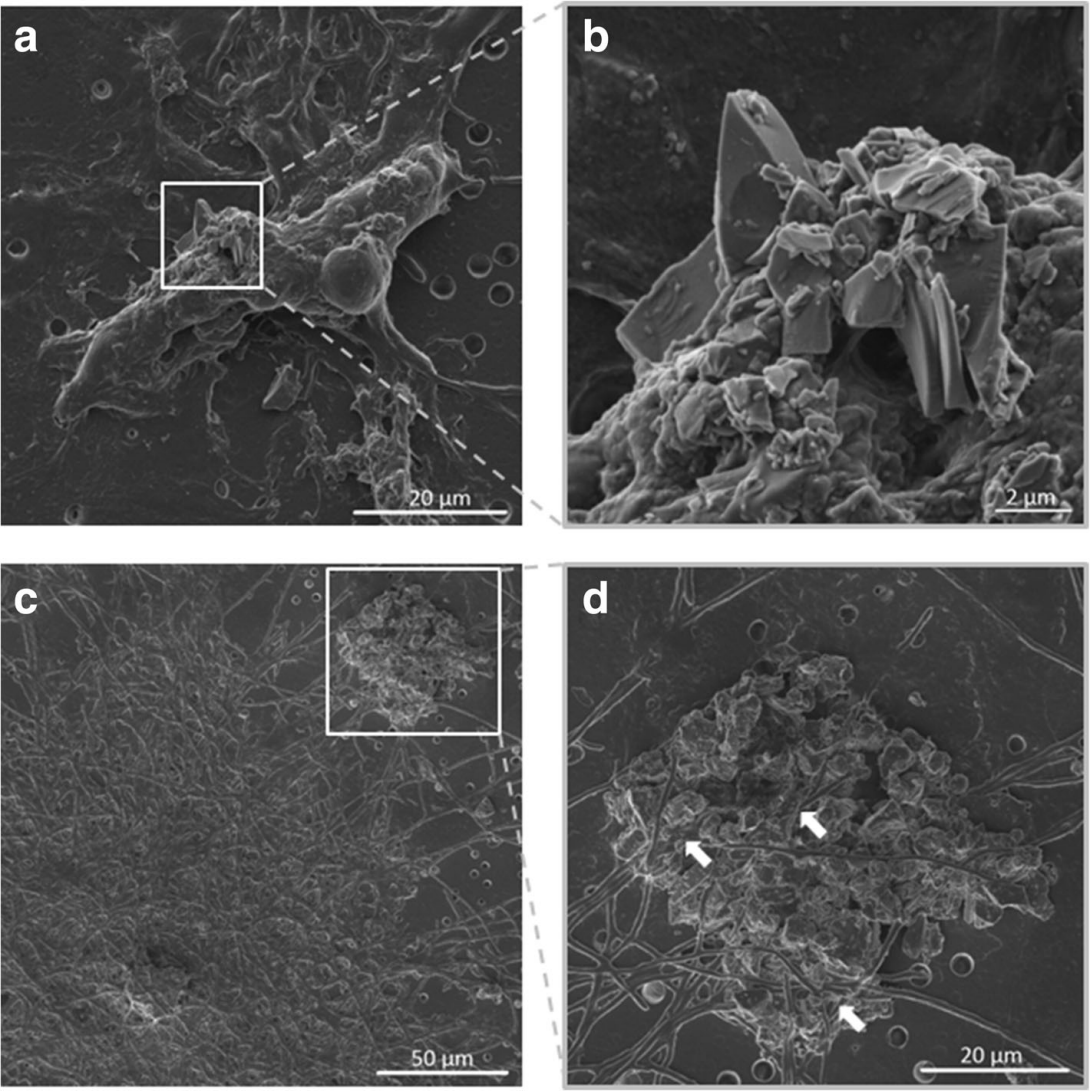
Interaction of volcanic ash with the triple cell co-culture. Scanning electron micrographs of the triple cell co-culture membrane exposed to 0.26 ± 0.09 μg/cm^2^ of single exposure to volcanic ash (SEVA), showing direct interaction of VA particles with the different cell types. **a** and **b** (inset of (**a**)) show a representative interaction of the human blood monocyte-derived macrophages (MDM) with VA, which appears to be engulfed by the MDM (**b**). Images (**c**) and **d** (inset of (**c**)) show a representative interaction of the human monocyte-derived dendritic cells (MDDC) with the VA particles, which are interacting with the pseudopodia of the MDDC (**d**; indicated with white arrows). Images were collected at 3 kV and 10 mm working distance. Scale bars are 20 μm (**a**), 2 μm (**b**), 50 μm (**c**) and 20 μm (**d**)

SEM images of the upper surface of the triple cell co-culture exposed to dry VA at the ALI showed that VA was able to directly interact with the macrophage cache of the co-culture system. Interestingly, VA particles were also observed on the basal layer of the triple cell co-culture, elucidative of a potential interaction with the MDDC present in this region (Fig. 4).

## Discussion

The purpose of this study was to gain a first understanding as to the potential hazard of a combined VA and DEP exposure to the respiratory system by using a state-of-the-art in vitro approach.

### Particle concentrations

For VA, concentrations of 0.26 ± 0.09 μg/cm^2^ (SEVA) and 0.89 ± 0.29 μg/cm^2^ (REVA) were chosen. It is difficult to state how representative these concentrations are in relation to ambient air concentrations following a volcanic eruption, due to the lack of reliable in vivo dosimetry data available and the fact that airborne volcanic ash concentrations are highly dependent upon the distance of a person from the volcano and the dynamics of the eruption itself. In addition, concentrations of respirable ash will be raised during ashfall but, also, later, due to resuspension by wind and human activity in dry conditions, and will dramatically reduce following rain, making average and cumulative exposures difficult to constrain. Searl et al. [21] measured PM_10_ on Montserrat during a period of frequent ashfall (1997–1999) from the Soufrière Hills volcano and found daily mean concentrations ranging from 0.05 to 1 mg/m^3^ when plentiful ash was in the environment, and 0.02–0.15 mg/m^3^ when there was little ash. It is also important to note that personal exposure to volcanic ash is highly influenced by activities undertaken by individuals as well as the general dustiness of the environment; hence concentrations associated with activities such as cleaning, or clearing the roads, may be higher than background concentrations, especially considering children [21, 22]. However, the general community will have lower personal exposures than the ambient levels as people will protect themselves (e.g. staying indoors) during ashfall, overnight and at times of high resuspension.

Assuming a daily inhaled air volume for humans to be 25 m^3^ and an alveolar lung surface area of *ca.* 100 m^2^, which correspond to a healthy, moderately-active adult [23], and an alveolar deposition efficiency of about 10% [24], it can be estimated [25] that the doses used in this study correspond to airborne concentrations which would not be encountered over a 24 h exposure relative to dry conditions of the highest ash concentration areas during the most active phase of the Soufrière Hills eruption [21]. Thus, from a hazard assessment approach, the doses used in the present study can be considered as a ‘worst-case’, or a particle overload scenario relative to such an exposure period to humans.

For DEP, a pseudo-ALI approach was adopted as it was not possible to aerosolize the hydrophobic DEP with the dry powder insufflator device, due to the electrostatic nature of DEP in dry powder form. For this reason, VA and DEP could not be applied concomitantly to the cell cultures as a dry powder mixture. Instead, as previously described in [26], a volume of 100 μl of DEP suspension in supplemented medium at 0.02 mg/mL was added to the cells grown on a 4.2 cm^2^ surface insert. Although these particles highly agglomerate, it was assumed that the majority of the DEP deposited on the cell surface at a dose of 0.5 μg/cm^2^.

Notably, the present study is a first screening of potential adverse effects of combined exposure to VA and DEP, therefore a simple approach of comparing the effect of a combined exposure with the effects of individual particles at comparable concentrations and exposure duration was used [27]. Importantly, the effects following exposures to individual particle types were considered, and single exposure data for DEP or VA are now compared to previous investigations using both in vitro and in vivo experiments.

### Single particle exposure biological effects

In the present study, DEP, alone, caused no significant (*p* > 0.05) cytotoxicity or oxidative stress to the co-culture. Previous studies also conducted with the same multicellular model have shown that DEP do not enhance the release of LDH, although it has been found that they induce oxidative stress [20, 28, 29]. Variation among studies can be attributed to differences in the applied exposure method [30, 31] as previously, the same co-culture as used in the present study was exposed to the same type of DEP (NIST 2975), albeit applied *via* suspension (in supplemented cell culture medium) to the apical chamber of the insert [20, 29], thus initiating different particle-cell interaction kinetics. In the present study, as previously mentioned, a pseudo-ALI approach was used, as previously described [26]. Notably, the lack of cytotoxicity and oxidative stress observed in the present study is contrary to previous findings using monocultures of these cell types [32–36]. This difference can be associated with the cellular interplay exhibited by this multicellular model, where two immune cell types (macrophages and dendritic cells) can directly interact with each other at the epithelium during reactions to particulate antigens [37], thus further highlighting the advantages of using multicellular models. Overall, though, it should be taken into consideration that different cell cultures, DEP compositions, the preparation of particle suspension and doses used in different studies vary, which makes a direct comparison amongst these studies challenging. Nonetheless, in the current study, DEP did cause an increased release of measured (pro-)inflammatory markers (TNF-α, IL-8 and IL-1β) compared to the negative control (supplemented cell culture medium). These findings concur with previous observations of monoculture in vitro studies, which reported DEP to be highly (pro)-inflammatory [38, 39], as well as with studies using the same triple cell co-culture system [20].

Ash from the Soufrière Hills volcano has been extensively studied over the past two decades [40] and is well characterised for its physical and chemical properties, including the sample used here [3, 41, 42]. The biological impact of Soufrière Hills ash has also received increased attention during this time [43–46], particularly due to the substantial crystalline silica present in ash derived from collapses of the Soufrière Hills lava dome (a pile of extruded, viscous lava sitting within the crater) [42]. Previous results from monoculture in vitro studies performed with Soufrière Hills ash are variable, due to the various and numerous different experimental designs employed and endpoints considered as well as the large amount of natural variability amongst ash samples [2, 46]. Despite this variability, Soufrière Hills ash is generally considered to be non-cytotoxic and have a low oxidative potential, but has the capacity to incite a limited (pro-)inflammatory response. Previous cell-specific studies on macrophages (PMA-differentiated THP-1 monocytes) and A549 epithelial 'like' cells indicated minimal cytotoxicity (measured by LDH release) and GSH depletion following exposures [46], however the response of other antigen presenting cells to VA particles has been largely, to date, uncharacterised. Similar findings have also been noted from in vivo studies [44, 47–49]. In this study, assessment of the biological response from the triple cell co-cultures following VA exposures alone resulted in no significant (*p* > 0.05) cytotoxicity, changes in cellular morphology, oxidative stress or release of (pro-)inflammatory mediators (TNF-α and IL-8). Therefore, the findings of the initial dose-respose analysis of VA exposures alone are largely in congruence with previous research with Soufrière Hills ash [43, 46, 50].

As evident from the discussion above, all previous in vitro studies performed on VA have used monoculture cell models, where the cell cultures have been immersed in cell medium and VA particles added, already suspended i.e. a pre-mixed sample, in liquid medium. Whilst this approach is commonplace, it does not adequately reflect the physiological condition of a respiratory exposure to VA as particles do not settle in the lung already immersed in liquid (which may affect the surface reactivity of the VA sample). The nebulisation method of VA using the dry powder insufflator (*DP-4, Penn Century, USA*) has enabled, for the first time for in vitro studies, application of VA to cells in its pristine, dry state. This method represents a more realistic scenario in comparison to all previous studies which used suspended ash in cell medium. The method has also shown good reproducibility, and can, therefore, be considered as a suitable method for conducting realistic in vitro respiratory hazard assessment of VA in future research activities. Furthermore, it has recently been shown that multicellular models can be useful tools in determining the specific (pro-)inflammatory and oxidative stress effects of particles compared to monocultures; as these models additionally take into account intercellular signalling among cells as it would occur in vivo [20].

The observation, by SEM, that the macrophages directly interacted with VA was expected, due to their role in the clearance of foreign material, and previous studies have shown the capacity of macrophages to internalise VA [46, 51]. The interaction with epithelial cells is not completely unexpected either, due to the surface area that they cover in the alveolar epithelial airway barrier in vitro system (insert membrane is 4.2 cm^2^ with 1 × 10^6^ epithelial cells seeded compared to 5 × 10^4^ macrophages seeded in the co-culture) [15, 37]. It is worth noting that, to the best of the authors’ knowledge, this was the first time that dendritic cells have been considered in terms of the biological impact of VA in vitro. The observed interaction of the VA with the MDDC can be hypothesized to occur either through (I) translocation of the VA particles *via* cell-cell interactions, as previously described for other particle types [37], (II) direct translocation through the pores of the micro-porous membrane insert (3 μm), or (III) deposition between the micro-porous membrane outer ridge and the side of the well of the six-well plate during the nebulisation process. Further research is therefore necessary to determine how VA becomes potentially available to interact, or not, with the dendritic cells of the co-culture system, such as through translocation studies previously performed with this 3D in vitro lung model [28, 52, 53], as well as to deduce what biological impact this interaction may potentially elucidate.

### Co-exposure biological effects

At sub-lethal concentrations, as with the SEVA, REVA and DEP alone, the combined exposures (DEP + SEVA and DEP + REVA) showed no significant cytotoxicity in the triple cell co-culture. By working in this concentration range, it was therefore possible to study further, mechanistic effects. The impact of any particle type upon the respiratory system is commonly associated with an increased level of oxidative stress [54]. Yet, in the current study, it was observed that no significant differences in oxidative stress levels were evident in any of the combined particle exposures compared to the negative control. In light of these observations, DEP showed no deviation from the negative control but VA treatment increased the relative abundance of GSH, an observation previously attributed to increased production by macrophages (in monoculture) to cope with volcanic ash [46]. Therefore, comparatively, the effect of DEP on GSH levels is greater than the effect seen with VA alone, and the effects noted with the combined exposure scenario could be attributed to the DEP driving an oxidative stress environment in the cell cultures rather than the absence of any oxidative stress. However, to elucidate the underlying mechanisms controlling oxidative stress levels in this combined exposure, further research is needed. Despite the lack of any cytotoxic and limited oxidative stress response following VA and DEP combined exposures, it was found that a heightened (pro-)inflammatory response occurred following exposure to respirable VA and DEP when applied as a combined exposure. Focussing firstly on TNF-α release, although there was a greater effect with the DEP + SEVA exposure than that of the individual response for both SEVA and DEP, the greatest response was observed following the combined exposure of DEP + REVA. It is difficult to state though whether this effect can be described as synergistic in comparison to the two particle treatments alone [27]. Furthermore, the impact of the DEP can also be seen in the combined exposure IL-8 response, as the DEP, DEP + SEVA and DEP + REVA responses are all raised, albeit not significantly (*p* > 0.05), in comparison to the negative control. Given that the apparent driver of the (pro-)inflammatory signal is DEP present in the exposure, this was further assessed by analysing the (pro-)inflammatory cytokine IL-1β. Release of this marker was significantly higher (*p* < 0.05) in the DEP + REVA scenario compared to the negative control and to DEP alone. The observed higher response of (pro-)inflammatory markers following exposure to the DEP + REVA compared to the DEP + SEVA scenario can be attributed to the effect of the greater combined dose of particles delivered to the cell surface.

In summary, the current study provides a first insight into the biological effects of combined exposure to VA and an urban pollutant (DEP) and implies a potentially greater hazard of simultaneously inhaling both particle types. The observations indicate that combined exposure to VA and DEP induces a (pro-)inflammatory response in cells at the respiratory epithelial tissue barrier, but it is not yet clear whether this effect is directly driven by the individual particle-cell interactions, secondary toxicology mechanisms incited *via* the particles’ physicochemical characteristics, or through particle-particle interactions leading to the combined effect noted. It is known that increased release of (pro-)inflammatory mediators may augment, as well as prolong, inflammatory reactions and, if the exposure persists, can result in chronic inflammation [38]. Airway inflammation not only promotes the development of lung diseases, but it may increase the susceptibility to acute cardiovascular disease [55]. Thus, the significance of these findings lies in the potential effects on respiratory health that this combined exposure may elucidate. These initial results are being used to inform future, planned work investigating chemical interactions between particles and the particle/gas/volatile mixtures of complete vehicle exhaust [56, 57] as well as volcanic emissions.

## Conclusion

The findings in the present study show that exposure to sub-lethal concentrations of VA with an urban pollutant (i.e. DEP) can promote a heightened and significantly increased (pro-)inflammatory response in vitro, absent of mediation by oxidative stress. The observed effects of the combined exposures are of further significance as, in some circumstances, they are greater than the response noted for DEP or VA, independently. It is envisaged that, in the event of future eruptions, the findings of this study will serve for a better understanding of the potential respiratory risk posed by combined exposure to urban pollution and VA towards human health. These findings will provide the basis for further investigations into the mechanisms driving the heightened (pro-)inflammatory response, in order to deduce the specific human health hazard, as well as how it may influence the respiratory function of susceptible individuals (i.e. with pre-existing lung diseases) in the population.

## Methods

### Chemicals and reagents

All chemicals and reagents were purchased from Sigma Aldrich (Switzerland), unless otherwise stated.

### Particle samples

#### Volcanic ash

##### Source

Ash from a dome-collapse ash-fall deposit of Soufrière Hills volcano, Montserrat (erupted and ash sample collected on 12 July 2003) was used (*MVO12/7/03*). The ash on Montserrat erupts into a very clean atmosphere (occasionally polluted by transfer of dust from the Sahara), so was chosen as a pristine example of ash which had no prior interaction with anthropogenic pollution. The bulk ash has previously been extensively characterised [3, 4, 41], and contains substantial quantities of respirable particles (cumulative volume % is 22.5 and 11.5 for <10 μm and <4 μm, respectively) [3] and is rich in crystalline silica (13.5 weight %) [4].

##### Sample preparation

A respirable fraction of VA was isolated using a Sioutas Cascade Impactor (*SKC Inc., USA*) and Leland Legacy sample pump (*SKC Inc., USA*) attached to a gravitational separation chamber. VA was introduced into an airstream established by operating the pump at a constant flow rate of 5 L/min. Aerosolised particles then entered the separation chamber where particles above a theoretical spherical aerodynamic diameter of 5 μm sedimented in accordance with Stoke’s law, calculated for a particle density of 1.0 g/cm^3^ in the system. These parameters have been empirically observed to produce an appropriate respirable-sized fraction in this set up. Remaining airborne particles were then sampled by the Impactor, which was assembled without impaction stage filters to enable sample recovery. Size-fractionated samples were then collated for use in characterisation and toxicity assays. Separation of the respirable fraction from the sedimented ash was conducted upon the same sub-sample on three different occasions, and then combined in order to maximise the recovery of respirable material needed for the study.

#### Characterisation of VA respirable fraction

Particle size distribution analyses of isolated respirable samples were performed using a Coulter LS230 (*Coulter Corporation, USA*) in water without sonication. Data were analysed according to the Mie theory of light scattering [58]. Results are the mean of three consecutive runs of the sample. Runs were 60 s each.

Surface area was determined using samples dried overnight at 105 °C according to the BET method [17], and analysed by nitrogen adsorption measurements at -196.15 °C using a Gemini III 2375 surface area analyser (*Micromeritics Instrument Corporation, USA*). Results are the mean of three independent measurements of the sample.

#### Nebulisation of VA respirable fraction

Respirable VA was nebulised over the cells using a dry powder insufflator (Model DP-4; *PennCentury Inc., Philadelphia, USA*). The use of the dry powder insufflator was based upon the method previously described by Bihari et al. [59]. Briefly, the ash was loaded into a sample chamber and then pushed through the device by small pulses of air administered to the device using a 10 mL commercial air syringe. The ash was discharged as a cloud from the end of a delivery tube and, in this way, nebulised over the cell culture plate located below the delivery tube within a closed nebulisation chamber. The quantification of deposited material was monitored by a QCM (with a detection limit 90 ng/cm^2^, AT-cut quartz, 5 MHz resonance frequency; *Stanford Research Systems, USA*) also located within the nebulisation chamber. Specifically, as material settles onto the QCM, the frequency of the crystal changes (ΔF). Calculated from the recorded frequency values before and after deposition of material, this ΔF value (Hz) is converted to deposited mass per area (μg/cm^2^) as described in [31]. In addition, as previously highlighted in [26], the deposition pattern can possibly change across each well of the six-well culture plate used. Analysis of the data showed that there was no difference in the deposition pattern across each well of the six-well culture plates used (data not shown).

##### Scanning electron microscopy

Nebulised respirable ash was imaged, uncoated, by a Mira3 LM (*Tescan, Czech Republic*) FE-SEM, using a secondary electron (SE) detector in order to visualise particle deposition and morphology.

#### Diesel exhaust particles

##### Source

Standard diesel exhaust particulate (DEP; NIST SRM 2975) was used. The key characteristics of this standard sample have previously been reported [14, 59].

##### Sample preparation

To produce a suspension of DEP, 1 mg of dry DEP was suspended in 1 mL cell culture medium RPMI 1640 (supplemented with 1% L-Glutamine, 1% Penicillin/Streptomycin and 10% fetal bovine serum). The pre-mixed solution was subsequently sonicated for 90 min at 37 kHz at 37 °C. This stock suspension of DEP was diluted with supplemented RPMI 1640 medium to a working concentration of 0.02 mg/mL.

### Hazard assessment

#### Lung cell cultures

All in vitro exposure experiments in this study were conducted with an established 3D triple cell co-culture model of the human epithelial tissue barrier cultured at the ALI [15, 52, 60]. This system has previously been described in detail [61]. Briefly, the model consists of a layer of human alveolar type II-like epithelial cells (A549) with human monocyte-derived macrophages (MDM) on the apical side (upper chamber) and monocyte-derived dendritic cells (MDDC) on the basal side (lower chamber). A549 epithelial cells were cultured at a density of 1 × 10^6^ cells per insert on BD Falcon cell culture inserts (high pore density PET membranes, 4.2 cm^2^ growth area, 3.0 μm pore size; *Beckton Dickinson AG, Switzerland*).

Human blood monocytes were isolated from different, individual buffy coats received from the Swiss blood donation service (Bern, Switzerland) (i.e. different donor for each exposure), using CD14^+^ MicroBeads as described previously [57]. Due to this, variations in the background among different sets of cell cultures were expected to occur. The cell culture densities of MDM and MDDC were 5 × 10^4^ cells/insert and 25 × 10^4^ cells/insert, respectively. Quantification of the cell-cell ratio for this co-culture system has previously been analysed and reported [37]. Co-cultures were incubated for 24 h under suspension conditions in order to allow cell-cell habituation. Subsequently cell culture medium was extracted from the apical layer to allow formation of the ALI over an extra 24 h period at 37 °C, 5% CO_2_ prior to particle exposures.

#### Lung cell exposures

##### VA exposures

In the approach used in this study, VA was administered as dry powder onto the upper surface of the co-culture at the ALI [37, 60] using a dry powder insufflator (Model DP-4; *PennCentury Inc., Philadelphia, USA*). Compared to the conventional particle suspension exposure, exposure at the ALI has been found to be a more sensitive in vitro exposure method, as it exhibits similar cellular responses at lower doses [30]. In addition, changes to the surface chemistry, morphology and size of the particles, which might affect the toxicological response of the system, are minimised.

As part of the initial dose dependent analysis to determine the optimal dose to use for VA in the combined exposure scenario, feed masses to the dry powder insufflator of 4, 6 and 8 mg of VA were used in a single dose exposure scenario. The corresponding dose that was deposited onto the cells was 0.13 ± 0.03, 0.21 ± 0.06 and 0.26 ± 0.09 μg/cm^2^ for 4, 6, and 8 mg starting (feed) mass, respectively. Additionally, repeated exposure to the highest dose (8 mg) was used in order to increase particle mass deposition onto the cells, as 8 mg was the feed maximum per nebulisation. A mass of 8 mg was loaded into the dry powder insufflator and nebulised over the cells three times within a time period of ~15 min. The average mass deposited in the repeated exposure scenario was 0.89 ± 0.29 μg/cm^2^.

##### DEP exposures

DEP were used in a pseudo-ALI exposure experiment, as previously described [26]. Briefly, a total volume of 100 μl of DEP at 0.02 mg/mL suspended in supplemented medium was added to the apical compartment of the triple cell co-culture model at the ALI, grown on a 4.2 cm^2^ surface trans-membrane insert. Upon assumption that the majority of the DEP would deposit homogeneously on the cell surface, the applied concentration would equate to a deposited concentration of 0.5 μg/cm^2^. It is important to note that this methodology was used due to the fact that dry DEP were found to be unsuitable for nebulisation using the dry powder insufflator due to their electrostatic nature as a dry powder (data not shown).

##### VA and DEP combined exposures

Directly after the exposure to DEP, the highest dose (chosen based upon the biological impact noted from the dose–response analysis and the efficiency of each dose deposited on the co-culture from the dry powder insufflator) of VA was applied, either as SEVA or REVA exposure, using the dry powder insufflator as previously described above in the section entitled ‘*Lung Cell Exposures; VA Exposures*’.

##### Post-exposure and sampling

Each exposure was followed by a 24 h incubation period at 37 °C and 5% CO_2_. After this, supernatants were collected and stored at either 4 °C or −80 °C until biochemical assays could be performed. In addition, insert membranes were fixed and prepared for immunofluorescent labelling or SEM microscopy, as described below.

### Biochemical assays

#### Cytotoxicity

##### LDH Release

Cytotoxicity, indicated by cell membrane damage, was determined by measuring the release of the intracellular enzyme lactate dehydrogenase (LDH) into the co-culture supernatant, assessed using an LDH cytotoxicity detection kit (*Roche Applied Science, Mannheim, Germany*) according to the manufacturer’s guidelines. The test was conducted in triplicate for each replication. The following repetitions for each exposure were conducted: SEVA *n* = 4; REVA, DEP, DEP + SEVA and DEP + REVA *n* = 3; negative and positive controls *n* = 8. Absorbance was determined at 490 nm after 10 min using a microplate reader (*Bio-Rad, Switzerland*), with a reference wavelength set at 630 nm. As a positive control, co-cultures were treated with 100 μl of 0.2% Triton X-100 in phosphate buffered saline (PBS) on the apical side and incubated for 24 h at 37 °C, 5% CO_2_.

##### Cell morphology

After the post-incubation period of 24 h, triple cell co-cultures were prepared for imaging *via* laser scanning microscopy (LSM). Cell membranes were fixed with 3% paraformaldehyde for 15 min at room temperature and then transferred to 0.1 M glycine in phosphate buffered saline (PBS) for 10 min. Samples were then washed x3 with PBS, and treated with 0.2% Triton X-100 for 15 min at room temperature to permeabilise the cell membrane for immunofluorescent staining. Subsequently, samples were stained with phalloidin-rhodamine (R-415; *Molecular Probes, Life Technologies Europe B.V., Zug, Switzerland*) in a 1:100 dilution to label the F-actin cytoskeleton, and with 1:100 dilution of 4′,6-diamidin-2-phenylindol (DAPI) at 1 μg/mL in 0.2% Triton X-100 + 1% BSA in PBS to highlight the cell nuclei. Visualisation of the samples was conducted with an inverted confocal LSM 710 (Axio Observer.Z1, *Carl Zeiss, Switzerland*) at a magnification of 63×. Representative images (z-stacks) were recorded at three independent fields of view for each sample (three independent samples were analysed (*n* = 3)) and were further processed using the 3D reconstruction software IMARIS (*Bitplane AG, Zurich, Switzerland*).

#### Oxidative stress

The total amount of reduced glutathione (GSH) in samples was quantified using a glutathione assay kit (*Cayman Chemical Company, Ann Arbor, Michigan, USA*) according to the manufacturer’s instructions. The detected concentrations of GSH are reported relative to the concentrations of total protein of each corresponding sample (determined by the Pierce BCA Protein Assay kit (*Pierce Protein Research Products, Thermo Scientific, Rockford, Illionis, USA*)), according to the manufacturer’s instructions. The negative control was cell culture medium only. As a positive control, co-cultures were treated with 250 μl of 100 mM tert-Butyl Hydrogen Peroxide (*t*BHP) on the apical side and incubated for 24 h at 37 °C, 5% CO_2_. For each replication, analysis was conducted in triplicate. The following repetitions for each exposure were conducted: SEVA *n* = 4; REVA, DEP, DEP + SEVA and DEP + REVA *n* = 3; negative and positive controls *n* = 8.

#### Chemokine/cytokine release

The (pro-)inflammatory response was investigated by quantifying tumour necrosis factor-α (TNF-α), interleukin-8 (IL-8) and interleukin-1β (IL-1β) release from the co-culture system into the basal cell culture well *via* enzyme-linked immunosorbent assays (ELISA DuoSet Development Kit, *R&D Systems, Minneapolis, Minnesota, USA*) according to the manufacturer’s protocol. The concentrations were determined spectrophotometrically at 450 nm using a microplate reader (*Bio-Rad, Switzerland*). Lipopolysaccharide (LPS, from *E-coli* at 1 μg/mL) was applied at a volume of 1.2 mL in the bottom compartment of the co-cultures and served as the positive control for TNF-α, IL-8 and IL-1β induction. The negative control was cell culture medium only. Analyses were conducted in triplicate for each replicate. The following repetitions for each exposure were conducted: SEVA *n* = 4; REVA, DEP, DEP + SEVA and DEP + REVA *n* = 3; negative and positive controls *n* = 8.

### VA:lung cell interactions in vitro

#### Scanning electron microscopy

Co-cultures exposed to SEVA were fixed with 3% paraformaldehyde for 15 min at room temperature and then sequentially washed with 20, 40 and 60% methanol for 5 min, 80% methanol for 3 min and washed 5 times with 100% methanol for 30 s. Samples were then dried in a vacuum desiccator over a 48 h period. Samples were then carbon coated and subsequently imaged with a Mira3 LM (*Tescan, Czech Republic*) FE-SEM, using an InBeam detector on a rotated stage (60°).

### Data and statistical analysis

All data are presented as the mean ± standard error of the mean, deriving from three individual experiments (*n* = 3) unless otherwise stated. All statistical analyses were performed using SPSS statistical software (IBM SPSS Statistics for Windows, Version 22.0, *Armonk, NY, USA*). Statistical significance was deduced through the use of a one-way analysis of variance (ANOVA), based upon normal distribution of the datasets. Subsequent Tukey’s *post hoc* tests were conducted to determine the specific statistical significance between the VA, DEP, DEP + SEVA and DEP + REVA exposures to the negative control (supplemented cell culture medium only). The alpha value was set at 0.05.

## Additional files

Additional file 1: Particle size distribution of the isolated respirable fraction of Soufrière Hills volcanic ash. Determined by a Beckman Coulter LS230 PSD analyser (*Coulter Corporation, USA*). Data are the mean of *n* = 3. (TIF 30 kb)
Additional file 2: Cell morphology and cytotoxicity of the triple cell co-culture exposed to different single exposure doses of volcanic ash. Confocal laser scanning microscopy (LSM) images show the F-actin cytoskeleton (red) and the nuclei (blue) of (a) control and cultures exposed to (b) 0.13 μg/cm^2^, (c) 0.21 μg/cm^2^, and (d) 0.26 μg/cm^2^ of respirable volcanic ash. Yellow arrows indicate cells undergoing cell division. Scale bars are 20 μm. Images were collected at magnification 63×. (e) Cytotoxicity as determined by the release of lactate dehydrogenase (LDH) from the triple cell co-culture following single exposure to 0.13 μg/cm^2^, 0.21 μg/cm^2^, and 0.26 μg/cm^2^ of respirable volcanic ash. Data are presented as fold increase relative to the negative control (cell culture medium only) ± standard error of the mean. Triton X-100 at 0.2% in phosphate buffered saline (PBS) acted as the positive assay control. LDH data shown are related to the following repetitions for each exposure: SEVA *n* = 4; negative and positive controls *n* = 8. (TIF 524 kb)
Additional file 3: Biochemical response of the triple cell co-culture following exposures to different doses of volcanic ash. (a) Total reduced glutathione (GSH), (b) tumour necrosis factor-α (TNF-α) release, and (c) interleukin-8 (IL-8) release of the triple cell co-culture model after single exposure to 0.13 μg/cm^2^, 0.21 μg/cm^2^, and 0.26 μg/cm^2^ of respirable volcanic ash. The respective positive assay controls are *tert*-Butyl Hydrogen Peroxide (*t*BHP; 250 μL of 100 mM) and lipopolysaccharide (LPS; 100 μL of 1 μg/mL), added to the apical and bottom compartment of the triple cell co-culture, respectively. The negative control was cell culture medium only. Data are presented as the mean ± standard error of the mean. Data shown are related to the following repetitions for each exposure: SEVA *n* = 4; negative and positive controls *n* = 8. (TIF 51 kb)

## Abbreviations

ALI: Air-liquid interface
BET: Brunauer-Emmett-Teller
DEP: Diesel exhaust particles
ELISA: Enzyme-linked immunosorbent assay
GSH: Total reduced glutathione
IL-1β: Interleukin-1 beta
IL-8: Interleukin-8
LDH: Lactate dehydrogenase
LPS: Lipopolysaccharide
LSM: Laser scanning microscopy
MDDC: Monocyte derived dendritic cells
MDM: Monocyte derived macrophages
NIST SRM: National Institute of Standards and Technology’s Standard Reference Material
PBS: Phosphate buffered saline
PM: Particulate matter
QCM: Quartz crystal microbalance
REVA: Repeated exposure volcanic ash
SE: Secondary electron
SEM: Scanning electron microscopy
SEVA: Single exposure volcanic ash
*t*BHP: *tert*-Butyl Hydrogen Peroxide
TNF-α: Tumour necrosis factor - alpha
VA: Volcanic ash
