# Supplementary figures and images for: Combined exposure of diesel exhaust particles and respirable Soufrière Hills volcanic ash causes a (pro-)inflammatory response in an in vitro multicellular epithelial tissue barrier model
Source: Part Fibre Toxicol. 2016 Dec 12;13:67. doi: 10.1186/s12989-016-0178-9 (PMC5153918; doi:10.1186/s12989-016-0178-9)

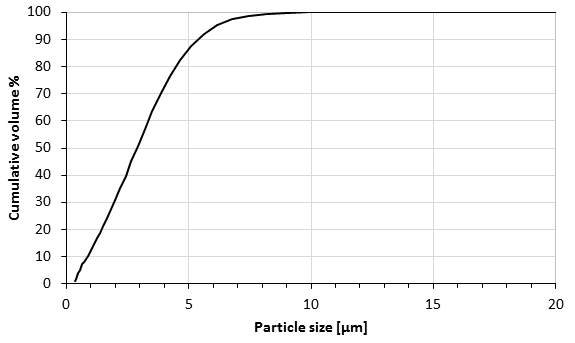

Supplement: Additional file 1: — Particle size distribution of the isolated respirable fraction of Soufrière Hills volcanic ash. Determined by a Beckman Coulter LS230 PSD analyser (Coulter Corporation, USA). Data are the mean of n = 3. (TIF 30 kb) [file 12989_2016_178_MOESM1_ESM.tif]

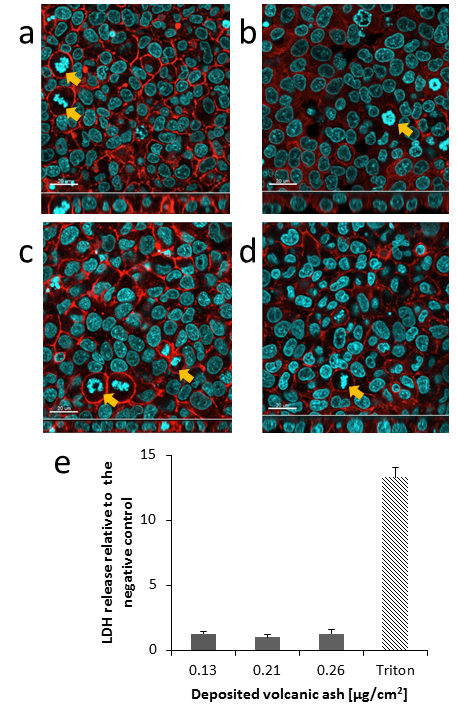

Supplement: Additional file 2: — Cell morphology and cytotoxicity of the triple cell co-culture exposed to different single exposure doses of volcanic ash. Confocal laser scanning microscopy (LSM) images show the F-actin cytoskeleton (red) and the nuclei (blue) of (a) control and cultures exposed to (b) 0.13 μg/cm2, (c) 0.21 μg/cm2, and (d) 0.26 μg/cm2 of respirable volcanic ash. Yellow arrows indicate cells undergoing cell division. Scale bars are 20 μm. Images were collected at magnification 63×. (e) Cytotoxicity as determined by the release of lactate dehydrogenase (LDH) from the triple cell co-culture following single exposure to 0.13 μg/cm2, 0.21 μg/cm2, and 0.26 μg/cm2 of respirable volcanic ash. Data are presented as fold increase relative to the negative control (cell culture medium only) ± standard error of the mean. Triton X-100 at 0.2% in phosphate buffered saline (PBS) acted as the positive assay control. LDH data shown are related to the following repetitions for each exposure: SEVA n = 4; negative and positive controls n = 8. (TIF 524 kb) [file 12989_2016_178_MOESM2_ESM.tif]

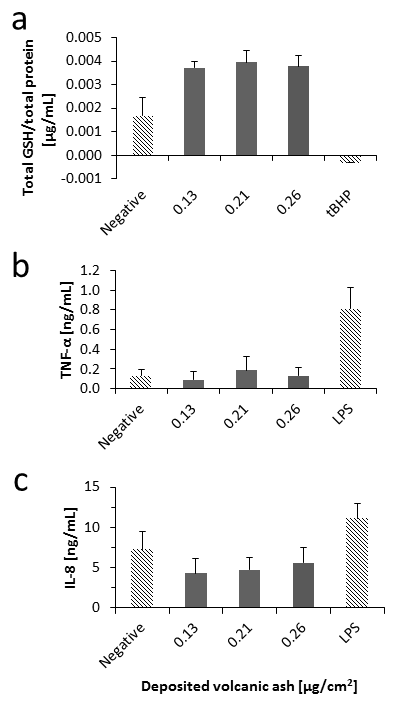

Supplement: Additional file 3: — Biochemical response of the triple cell co-culture following exposures to different doses of volcanic ash. (a) Total reduced glutathione (GSH), (b) tumour necrosis factor-α (TNF-α) release, and (c) interleukin-8 (IL-8) release of the triple cell co-culture model after single exposure to 0.13 μg/cm2, 0.21 μg/cm2, and 0.26 μg/cm2 of respirable volcanic ash. The respective positive assay controls are tert-Butyl Hydrogen Peroxide (tBHP; 250 μL of 100 mM) and lipopolysaccharide (LPS; 100 μL of 1 μg/mL), added to the apical and bottom compartment of the triple cell co-culture, respectively. The negative control was cell culture medium only. Data are presented as the mean ± standard error of the mean. Data shown are related to the following repetitions for each exposure: SEVA n = 4; negative and positive controls n = 8. (TIF 51 kb) [file 12989_2016_178_MOESM3_ESM.tif]
